# ‘Your health our concern, our health whose concern?’: perceptions of injustice in organizational relationships and processes and frontline health worker motivation in Ghana

**DOI:** 10.1093/heapol/czu068

**Published:** 2014-09-11

**Authors:** Matilda Aberese-Ako, Han van Dijk, Trudie Gerrits, Daniel Kojo Arhinful, Irene Akua Agyepong

**Affiliations:** ^1^Ghana Health Service, Navrongo Health Research Centre, P.O. Box 433, Bolgatanga, UER, Ghana, ^2^Sociology and Anthropology of Development Group, P.O. Box 8130, 6700 EW, Hollandseweg 1, Wageningen, The Netherlands, ^3^Graduate School of Social Sciences, Kloveniersburgwal 48 1012 CX Amsterdam, University of Amsterdam, The Netherlands, ^4^Noguchi Memorial Institute for Medical Research, NMIMR, University of Ghana, P.O. Box LG 581 Legon and ^5^School of Public Health, University of Ghana, P.O. Box LG 13 Legon

**Keywords:** Attitude, frontline health workers, Ghana, justice, motivation, people-centred health systems

## Abstract

Taking a perspective of frontline health workers as internal clients within health systems, this study explored how perceived injustice in policy and organizational matters influence frontline health worker motivation and the consequent effect on workers’ attitudes and performance in delivering maternal and neonatal health care in public hospitals. It consisted of an ethnographic study in two public hospitals in Southern Ghana. Participant observation, conversation and in-depth interviews were conducted over a 16-month period. Ethical approval and consent were obtained from relevant persons and authorities. Qualitative analysis software Nvivo 8 was used for coding and analysis of data. Main themes identified in the analysis form the basis for interpreting and reporting study findings. Findings showed that most workers perceived injustice in distributive, procedural and interactional dimensions at various levels in the health system. At the national policy level this included poor conditions of service. At the hospital level, it included perceived inequity in distribution of incentives, lack of protection and respect for workers. These influenced frontline worker motivation negatively and sometimes led to poor response to client needs. However, intrinsically motivated workers overcame these challenges and responded positively to clients’ health care needs. It is important to recognize and conceptualize frontline workers in health systems as internal clients of the facilities and organizations within which they work. Their quality needs must be adequately met if they are to be highly motivated and supported to provide quality and responsive care to their clients. Meeting these quality needs of internal clients and creating a sense of fairness in governance arrangements between frontline workers, facilities and health system managers is crucial. Consequently, intervention measures such as creating more open door policies, involving frontline workers in decision making, recognizing their needs and challenges and working together to address them are critical.

**KEY MESSAGES**

- Frontline health workers perceive that they do not receive ‘people-centered care’ from their employers, despite being asked to provide ‘people-centered care’ to the clients that come to health facilities. This considerably weakens the credibility of the message they are being given to treat their clients in a responsive manner.
- They perceive procedural, distributive and interactional injustice at policy and organizational levels, which have a strong influence on worker motivation and response to client health care needs.
- Health workers’ quality needs must be adequately met if they are to be adequately motivated and supported to provide high quality and responsive care to clients they interact with on a daily basis.
- An important dimension to meeting these quality needs of frontline workers is real and perceived justice in governance arrangements that puts a human face to interactions between frontline workers and their facility and health system managers such as creating more open door policies, involving frontline workers in decision making, recognizing their needs and challenges and working together to address them is crucial.

## Introduction

Policy makers and other agents responsible for reforming African health institutions and systems have often blamed health workers for a poorly responsive health system, suggesting that health workers interact and communicate poorly with clients (Agyepong *et al.* 2001; Ministry of Health 2001; Andersen 2004; Ministry of Health 2007). Interventions to improve quality and responsiveness in healthcare have centred on professionals and frontline workers without recourse to a total system reform (Agyepong *et al.* 2001). Yet, low health worker motivation and discontent continue to be cited as major causes of poor healthcare quality and outcomes in Sub Saharan Africa including Ghana (Agyepong *et al.* 2004; Luoma 2006; Chandler *et al.* 2009; Adzei and Atinga 2012; Agyepong *et al.* 2012; Alhassan *et al.* 2013; Faye *et al.* 2013). Worker motivation can be defined as the degree of willingness of the worker to maintain efforts towards achieving organizational goals (Kanfer 1999; Franco *et al.* 2002). Extrinsic motivation factors including contingent rewards such as salary, policy reforms and organizational factors and intrinsic motivation factors that embody the individual’s desire to perform the task for its own sake, which is self generated and non-financial such as interpersonal factors have been cited as influencing worker motivation in Africa including Ghana (Agyepong *et al.* 2004; Andersen 2004; Chen *et al.* 2004; Rowe *et al.* 2005; Ansong-Tornui *et al.* 2007; Bosu *et al.* 2007; Witter *et al.* 2007; Willis-Shattuck *et al.* 2008; Mbindyo *et al.* 2009; Songstad *et al.* 2011; Prytherch *et al.* 2012; Mutale *et al.* 2013). Thus, worker motivation is an important indicator of the quality and responsiveness of an organization towards its frontline health workers.

Continuous quality improvement (CQI) is a management philosophy as well as approach. It is a philosophy in that it has underlying beliefs, ways of thinking, concepts and attitudes about quality improvement. From a CQI philosophical perspective, quality is the product of a chain in which each person is a customer (client) of the people in the process preceding theirs (McLaughlin and Kaluzny 1994; Agyepong *et al.* 2001, 2004). The external users of the services of a particular organization e.g. the mother who brings her child for an immunization or the woman who comes to deliver at a health facility, who in the health system are called clients or patients are the last in the chain. The quality and responsiveness of the service they receive will be influenced by the quality and responsiveness of the whole customer chain, which starts at the top of the organization and ends with them. In this conceptualization, the workers in an organization are seen as internal customers or clients and the clients at the end of the chain are the external customers or clients. For example, if the administration office has delayed a nurse’s request for better conditions of service or supplies, she may become irritated and frustrated and the chances that she will have a negative attitude towards her work increases, which in turn will influence her response to her clients (external customers of the organization). The CQI philosophical concept of internal and external customers of an organization may be a more inclusive concept to use in thinking through how to make health systems people centred. The nurse in our illustrative example has not received ‘people-centred care’ from her organization, which may negatively affect her ability to deliver ‘people-centred care’ to the clients (external customers) who have come to her. People-centred care has been defined as:

“….care that is focused and organized around people, rather than diseases. Within a people-centred approach, disease prevention and management are seen as important, but are not sufficient to address the needs and expectations of people and communities. The central focus is on the person in the context of his or her family, community, and culture (WHO 2011).”

Drawing upon the CQI philosophy related to internal and external customers, if quality is the end result of a linked chain from internal through to external customers; then for an organization to function well and provide quality care to its clients, it has to take care of the quality needs of its workers or internal customers. This study sought to explore frontline health worker experiences and perceptions of justice in national and organizational policies, processes and procedures relevant to their work; and how these issues influence their motivation and responsiveness to clients in the provision of maternal and neonatal health care. The study answered the questions: How do frontline health workers perceive justice (fairness) in the support they receive from the organization they work for and how does that influence their motivation to respond to their clients’ health care needs? To explore the various dimensions of worker experiences organizational justice theory has been employed.

Organizational justice theory is one of the critical theories in studying worker motivation (Latham and Pinder 2005; Zapata-Phelan *et al.* 2009; Songstad *et al.* 2011). Justice and fairness are concepts with similar meanings and in this paper will be used interchangeably. Both concepts have to do with impartiality, reasonableness, justice and equity (Agyepong 2012). Organizational justice is used to pinpoint the individual’s belief that the distribution of outcomes, or procedures for distributing outcomes such as pay and other opportunities are fair and appropriate when they satisfy certain criteria (Leventhal 1976; Bell *et al.* 2006). The theory is relevant to this study because perceptions of justice have been known to elicit different behavioural reactions including positive or negative attitudes in worker response to work demands and performance within organizations (Greenberg 1993; Konovsky 2000; Laschinger 2003; Colquitt *et al.* 2006; Zapata-Phelan *et al.* 2009). When workers perceive injustice they may become demotivated and repay the organization with negative attitudes, which affects organizational climate. Where they perceive fairness they are more inclined to be motivated and repay the organization with positive attitudes including trust and positive response to organizational and clients’ needs (Cropanzano *et al.* 2002).

We theorized that a frontline health worker’s judgement of fairness in policy and organizational processes elicits reactions that influence motivation and response towards work, which affects the worker’s desire to perform tasks that contributes to the achievement of organizational goals. This makes organizational justice an appropriate concept for exploring processes that shape health worker motivation and response to clients’ needs in a hospital context.

The idea of organizational justice is based on Leventhal’s two-dimensional distinction of procedural and distributive justice (Leventhal 1976) and interactional justice (Konovsky 2000; Colquitt *et al.* 2001). Procedural justice is defined as an individual’s belief that allocative procedures or decision-making processes, which satisfy certain criteria are fair and appropriate (Leventhal 1976; Cropanzano *et al.* 2002). Distributive justice is perceived as the individual’s belief that it is fair and appropriate when outcomes or rewards such as salary, punishments or resources are distributed in accordance with certain criteria (Leventhal 1976; Colquitt *et al.* 2001; Stinglhamber *et al.* 2006; Cropanzano *et al.* 2002). Interactional justice has been defined as the quality of interaction between individuals (Cropanzano *et al.* 2002; Stinglhamber *et al.* 2006). Interactional justice contains two aspects, informational and interpersonal justice. Informational justice is defined as the extent to which individuals are provided with information or rationale for how decisions are made (Greenberg 1993; Laschinger 2003; Almost 2006). Interpersonal justice is defined as the extent to which individuals are treated with respect and dignity (Greenberg 1993; Laschinger 2003; Almost 2006).

All three dimensions of justice distributive, procedural and interactional justice will be used in this study to explore workers’ perceptions of justice in policy and organizational processes within the hospital context, as they were evident in worker narratives. Although distributive justice focuses on the final outcome, procedural justice deals with the processes involved in arriving at the final outcome (Leventhal 1976). The line between the two can be very thin, and in our findings some of the issues presented had both procedural and distributive justice complexly interrelated, so the two dimensions of justice will be discussed concurrently.

## Methods

Health worker motivation has been widely studied using a variety of qualitative (Dieleman *et al.* 2003; Dieleman *et al.* 2006; Bradley and McAuliffe 2009) and quantitative (Franco *et al.* 2004; Purohit and Bandyopadhyay 2014) methods. To reflect the complex nature of factors influencing health worker motivation in Africa including Ghana (Hongoro and Normand 2006), an ethnographic study was conducted in two public hospitals in Southern Ghana. Ethnographic studies provide ‘thick description’ (Geertz 1973) and rich details of social phenomena. Additionally, they provide voice to those such as frontline workers whose experiences receive little attention (Fahie 2014). This method requires long and active periods in the site of study to learn, experience and represent the lives of subjects in their natural setting (Van der Geest and Sarkodie 1998; Emerson *et al.* 2005). Consequently, M.A. referred to as ‘the researcher’ worked as a student researcher in the two hospitals over a 16-month period as part of her PhD thesis research. She employed ethnographic methods including participant observation, conversation and in-depth interviews to collect data among health workers in the hospitals. As an active participant in the process of health care provision, the researcher observed how motivation and demotivation is produced through worker interaction with their environment.

For purposes of anonymity, the hospitals are referred to as Facility A and Facility B and pseudonyms are used for all names used in this article. Facility A serves a metropolitan area with a population of about half a million. It has specialist units, services, as well as workers including obstetrician gynaecologists, anaesthetists and paediatricians. It provides comprehensive inpatient care with a bed complement of 294. It has a theatre that permits major surgical operations and the full range of emergency obstetric services in addition to routine delivery services. Facility B serves a peri-urban population of about 200 000 inhabitants. It has a bed capacity of 20 and provides only basic maternity services. It had no theatre for major surgical operations during the period of study, but efforts were being made to set up one. The facility refers complicated obstetric and gynaecological cases needing specialized services to better-equipped facilities outside the district. Its doctors are general practitioners.

Facility A was selected to help gain insight into the study questions in the context of a big specialist hospital. Facility B was chosen to help understand the same issues in a smaller non-specialist hospital. Data were collected in two phases. M.A. collected data in the maternity and new-born units of Facility A from January to September 2012 and in the maternity department of Facility B from October to December 2012. In the second phase, she collected data in Facility B in July and August 2013 and in Facility A in October and November 2013. Table 1 gives a breakdown of categories of workers and the methods used to obtain data. Data were collected on task agreement, relationships between professional groups and management, challenges and benefits in health care provision, trust relations and motivation. Attitudes and workers’ response to clients’ needs were observed by the researcher as well as crosschecked with health care providers.

**Table 1:** Categories of workers in Facilities A and B who were included in the study and methods used in collecting data

Category of workers	Data collection methods	
	Conversation	Interviews
**Facility A****^a^**		
Nurses and midwives	62	12
House officers	5	2
Senior doctors	11	4
Anaesthetists	5	3
Ward aids	2	2
Orderlies	6	6
Doctors who left Facility A	—	2
Laboratory officials	—	2
Departmental supervisors	9	1
Facility management workers	3	4
**Facility B****^b^**		
Nurses and midwives	23	7
Nurse who left the facility	1	1
Doctor	1	1
Ward aids	4	—
Departmental supervisors	3	4
Facility management workers	2	4

^a^In Facility A observation was carried out in the antenatal and postnatal clinics, labour, lying in and the gynaecological wards and the maternity theatre. Additionally, the ethnographer participated in meetings, doctors’ ward rounds, training and workshops for workers.

^b^In Facility B observations were done in the antenatal and postnatal clinics, the labour ward and the hospital pharmacy. Also, the ethnographer participated in district annual performance review and a party for five retirees.

Notes from observation of events, participation in workshops among others and conversations were jotted down in field note books. The notes were reconstructed and expanded at the end of each field visit in line with standard ethnographic studies (Emerson *et al.* 2005). Interviews were tape recorded and transcribed verbatim by a neutral researcher. The aim of employing a neutral researcher was to preserve interviewees’ original expressions and to enhance validity of the study. Observation notes, conversations and transcribed interviews were typed and transferred to qualitative analysis software Nvivo (version 8), which was used to generate a coding list on common themes that arose from the data. Subsequently, the data were systematically analysed to identify patterns, differences and contradictions. Secondary data including institutional reports, policy guidelines and circulars were used to support and crosscheck the findings.

Main themes identified were related to distributive, procedural and interactional justices at local hospital management and the wider health sector decision-making levels. These three dimensions of justice form the basis for interpreting and reporting on study findings at the two levels. Additionally, intrinsic motivating factors were found and they are also discussed. While different categories of frontline workers were studied, the findings focuses on doctors, nurses and anaesthetists’ experiences, because these three categories of frontline workers are tasked with the core responsibility of providing maternal and neonatal health care.

## Findings

The researcher participated in a workshop that was organized by the management of Facility A for selected health workers (administrators, doctors, nurses, paramedics) at the facility. The objective of the workshop was to improve workers’ knowledge on legal issues concerning the rights of workers and clients. Towards the close of the workshop workers were given the opportunity to ask questions. The excerpts below of a question a nurse–administrator asked a facilitator who is a doctor and also a frontline worker and the response shows in a nutshell perceived policy and organizational injustice issues encountered by nurses, doctors and anaesthetists in everyday health care provision in Facility A as indeed was also the case in Facility B, where subsequent fieldwork was conducted.

“Nurse: We have been talking about how to attend to clients for two days, what do you have for us, health workers?

Facilitator: It is shameful that companies pay for their workers who we take care of. But in health institutions we who take care of them pay our own medical bills. ‘Your health our concern, our health whose concern?’ That is why they believe health workers steal things. In those institutions they reimburse health bills. Why do you think you should use all the internally generated funds (IGFs) for services and not to take care of yourselves? You think VALCO and Electricity Company of Ghana use all their money to buy steel and electricity! They use some to take care of their workers.”^1^

The interaction suggests that health workers perceive that the values they are being asked to hold for their clients are not the values they feel are being held for them as people in the health system by their employers.

First, the nurse’s question suggests perceived neglect of frontline workers, who are yearning for attention. Second, the facilitator presents layers of perceived injustice confronting health workers. He suggests injustice in policy regarding conditions of service of health workers compared with their colleagues in other establishments. He also brings out organizational matters including interactional injustice regarding a common negative perception that health workers are thieves who steal medical supplies from public hospitals to sell to private hospitals. Additionally, he brings out issues of distributive injustice on how monies generated by health workers within their facilities are used. He suggests that the electricity company that supplies most parts of the country electric power and VALCO company, which produces aluminium derived from bauxite of world-class quality to meet local demand and for export, are ‘people centred’, because they use their companies’ revenue to purchase raw materials for production to meet their external customers’ electric power needs and equally use part of it to take care of their internal ‘customers’’ health needs. He juxtaposes the Ghana Health Service (GHS) logo: ‘Your health our concern’, which suggests that the health of the external customer is the responsibility of the health worker with ‘Our health whose concern?’: implying that the health worker’s health needs are not the responsibility of anyone.

**Figure 1:**
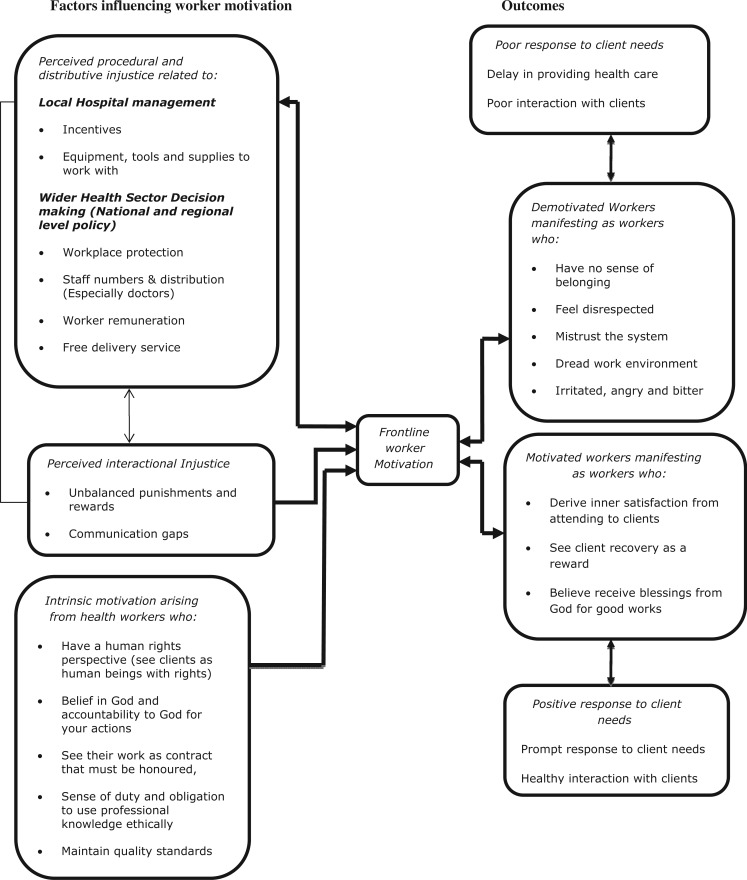
Processes in health worker motivation.

Thus, health workers who are the custodians of health care of the general public perceive that they do not receive ‘people-centred’ care. This interaction fits Ntim’s assertion in his article on economic governance and social accountability in Ghana: ‘The moment there is a perception of unfairness—that others are having more than their due, this de facto precipitates agitation*’* (Ntim 2013). This goes to support other findings in this study that suggest that majority of frontline health workers perceive distributive, procedural and interactional injustice to be operating at local hospital management and the wider health sector decision-making levels. By the wider health sector decision-making level, we are referring to the Ministry of Health as well as its national directorate; the GHS and the regional-level directorates, which have the responsibility for making decisions that become authoritative for the lower levels (districts, hospitals and below). In the rest of this section the findings will present narratives of frontline workers based on Figure 1 as follows: distributive, procedural and interactional injustice at hospital management and the wider health sector decision-making levels. Factors influencing intrinsic motivation of frontline workers and consequences on workers’ response to clients’ needs will also be discussed.

## Perceived procedural and distributive injustice related to local hospital management

Frontline workers perceived distributive injustice by hospital management in the provision of incentives and response to equipment, tools and supplies to work with and their infrastructure needs, which are discussed below.

Workers in Facility A said in past times they were given incentives such as a monthly transport allowance and a Christmas package. However in recent times management had failed to provide these incentives, which they considered unfair.^2^ They suggested that it had contributed to a reduction in worker motivation to respond to clients’ needs. In the words of a frontline worker:

“I think the problems are coming from here. Last two years when they decided not to motivate us at Christmas, they thought we will talk, so the director quickly went on leave….People are not complaining because they are all smart and finding their way around by doing their own things.”^3^

Additionally, interviews and conversations with nurses, anaesthetists and doctors including some doctors who had left Facility A, suggested that they perceived that management did not treat doctors posted to the maternity department fairly. So they were not motivated to stay. One of the doctors who had left the facility stated: ‘I thought ‘I will be given accommodation at Facility A’, but they denied me. I thought after that ‘they will give me some allowance for fuel’; no they denied me.’^4^

In response, a management worker said that the facility stopped providing incentives to workers because a directive from the director general’s office in 2008 ordered all facilities to stop issuing incentives.^5^ Some frontline workers indicated that they were aware of the directive. Nevertheless they argued that their output is high, which enables the hospital to generate a lot of revenue. So, it was only fair that they should be appreciated for their efforts by being given monetary incentives.^6^

On the issue of doctors leaving Facility A, the facility manager responded that the facility had recently introduced an incentive package specially for doctors in the maternity department to help maintain the few doctors that were in the department.^7^

In Facility B, midwives complained of lack of incentives including the provision of drinking water, infrequent allocation of Christmas bonuses and stoppage in providing night cups (coffee, tea and biscuits) for workers on night duty.^8^ In response, two management workers explained that management in consultation with frontline workers agreed to sacrifice all incentives to workers and rather use the money to buy essential items, which were required for a peer review^9^ exercise. They said that all frontline workers agreed to sacrifice and were happy about it.^10^ Conversations with midwives on night duty, however, suggested that they were not aware of this arrangement.^11^

Some workers in Facility A bemoaned deteriorating conditions of the hospital’s infrastructure resulting in some injuries to workers. For instance during a maternal audit meeting a senior nurse and a senior doctor narrated how a theatre door fell on a nurse. No compensation was provided to the nurse afterwards. They suggested that it was not fair that though their efforts brought in money management did not provide them with a conducive work environment.^12^ A frontline worker summed the situation up:

“All that we are asking is every day we work, but where does the money go? Look at the air conditioners and the fans on the wards, they are not working! But when you go to their offices (management workers) you will see that everything works.…Yet, those of us who do the real work and bring in the money, you come to our offices and we are crammed and nothing works.”^13^

In both hospitals, frontline workers perceived procedural injustice in their respective hospitals management response to their equipment and basic medical supplies needs. Some added that sometimes they were not involved in decisions to acquire supplies and equipment, for which they are the end users. Also whenever they were involved their views were not taken into consideration.^14^ They felt that the hospitals delayed in providing them with basic supplies and sometimes they were given substandard products to work with. They perceived these acts as unfair to frontline workers who have to improvise on such occasions to provide health care to clients. They argued that this contributed to delays in providing services to clients. Some said using substandard products contributed to the provision of poor quality care to clients. They indicated that poor response by managers to provide their working essentials was demotivating.^15^

Management workers on the other hand responded that the seemingly poor response to supplies and equipment needs was because facilities are required to follow procurement laws for bulk purchases. Unfortunately, the procurement process takes some time and that accounts for the delay.^16^ For substandard medicines and other supplies, they admitted that this was a challenge to management as well. Facilities are by law not allowed to buy supplies and equipment from the open market if the Central or Regional Medical stores have some in stock. Yet, sometimes medicines issued to facilities from the Central medical stores are expired or fake. To support this assertion, two management members cited an occasion that Facility B returned quantities of oxytocin,^17^ which the medical stores supplied to the hospital, because they were discovered to be fake.^18^

The majority of workers indicated that management’s inability to provide incentives, the needed medical supplies and failure to maintain safety standards was demotivating and a sign of management’s lack of appreciation of their work. Thus they did not trust that management was working in workers’ interests.^19^ This supports Adzei and Atinga’s (2012) study, which suggests that resources to work with and the quality of hospital infrastructure are significant determining factors of health worker motivation and retention in district hospitals in Ghana. Other studies equally suggest that health workers’ inability to pursue their vocation due to lack of means and supplies is a demotivator (Mathauer and Imhoff 2006). Also related to this finding but in a contrary direction procedural justice has been found to lead to increased job satisfaction, organizational commitment and organizational citizenship behaviour (Konovsky 2000). Thus, workers’ perception of injustice was observed to have contributed to a lack of commitment and anti-citizenship behaviour that was counterproductive to the achievement of organizational goals. We observed that in part at least, as a consequence of these perceptions that the organization was not interested in their welfare as people, there was low worker motivation that had led to attitudes that created tensions and contributed to poor organizational climate and poor worker collaboration in health care provision. Ultimately, it affected worker response to clients’ needs. Some workers had adopted strategies including doing locum^20^ in private facilities, charging clients illegal fees or reporting to work late or leaving work early.^21^ Sometimes such attitudes led to delays in responding to clients’ needs, due to poor collaboration among different categories of professionals providing services such as Caesarean sections (CS). An illustration is a junior doctor who had to wait for nurses, orderlies and an anaesthetist on afternoon shift to arrive to work with him to perform a CS on a client who was admitted the previous day and needed an emergency CS. In response to a question on factors demotivating him he expressed his frustration as follows:

“Look at that woman lying there (a pregnant woman set with infusion is lying on a bed in the walkway), she has been in labour since Sunday (this was a Monday afternoon), but now we cannot perform CS on her, because it is 1:30 pm and the morning shift people say they have to close.”^22^

Ideally the morning shift should have worked with the doctor till the afternoon shift took over at 2.00 pm. This kind of situation has been observed elsewhere (Mansour *et al.* 2005; Heponiemi *et al.* 2010). Still related to these observations, but in the contrary direction, other studies have found that where workers had trust in management, it reflected in a positive relationship between workers and their clients (Bruce 1990; Koenig *et al.* 1997; Westaway *et al.* 2003; Atinga *et al.* 2011).

### Perceived distributive injustice related to wider health sector issues at national level

Folger (1993) suggests that when employees perceive that their organization cares about them as human beings, they are more likely to trust the organization, exhibit greater loyalty and commitment to work and the contrary is true. Many of the frontline workers in this study perceived injustice at a wider health sector level that is the central Ministry of Health, GHS and its regional health service directorates. They suggested that the sector was not responsive to their health care needs, work-related injuries and providing them with a conducive work environment. Frontline workers’ perceptions of injustice at sector level sometimes intersected with their perceptions of injustice at hospital management level.

Frontline workers suggested that the Ministry of Health, GHS and their facility managers did not care about their welfare. Consequently, they did not trust that GHS and their facilities would take care of them if they risked their lives in the line of duty. Frontline workers’ lack of trust was sometimes exhibited in worker–client interaction. The observation below is an illustration of one of such incidents in a maternity ward. A mentally challenged client was in labour, but she was not co-operating with a senior nurse, who wanted to conduct a vaginal examination. A junior nurse discouraged the senior nurse from continuing her efforts by saying:

“If she will not agree…. leave her.…If you force to examine her and she resists, you could injure yourself…. Ghana Health Service will not do anything for you. You will even have to take care of yourself, buy your own drugs, treat yourself and no one will compensate you.”^23^

Interviews with management workers suggested that there was a work policy guideline for adverse events to ensure that workers who got injured were catered for.^24^ However, workers who were injured or exposed to HIV/AIDs and Hepatitis B in the process of providing health care said they had to bear the cost of treatment. A doctor in Facility A who experienced needle pricks on three occasions while performing surgery on HIV/AIDS clients said he had to pay for the cost of treatment.^25^ A nurse in Facility B also narrated her experience as follows:

“If a worker is sick even paracetamol (a painkiller usually administered as first aid) you have to buy…Last year I was doing delivery and had to do episiotomy. While I was suturing, I suffered a needle prick. Unfortunately, the client was hepatitis B positive….I had to do some tests… I also had to go for hepatitis B vaccination and the disease control officer charged me 15 Ghana Cedis (US$7) for each of the three shots.”^26^

The researcher interviewed a legal expert to understand whether workers had a right to demand treatment for injuries at work and better conditions of service. He said that the Ghana labour act stipulates that the health of the employee is the concern of the employer. So workers had the right to demand better conditions of service. He added that it was more rewarding to the organization to provide such basic services to their frontline workers, because it served as a booster to worker performance.^27^

Frontline workers in Facility A perceived distributive injustice from the regional health directorate and hospital management in the allocation of frontline workers especially doctors to the maternity department of Facility A. Facility A conducts over 200 deliveries in a week. At the time of the field work, it had three specialist obstetrician gynaecologists and three general doctors. Additionally, an average of three house officers (newly qualified doctors on internship) were posted to Facility A’s maternity department periodically to do 3- to 6- month internship under the supervision of specialists. Doctors complained of unfair distribution of doctors and work between them and their colleagues in the teaching hospitals. They suggested that in comparison, the teaching hospitals attended to only a slightly higher number of maternity cases than they did, yet had about seventy doctors in their maternity departments compared with the six in Facility A’s maternity department.^28^

Some suggested that the regional health directorate was unresponsive to their need for doctors, despite efforts put in by the maternity department to bring their predicament to its notice. Conversation with some doctors in the maternity department and an interview with a doctor who left the facility suggested that an assessment of the quantum of work by the regional health directorate recommended that the maternity department be staffed with 25 doctors. But the regional health directorate did not provide the recommended number of doctors. They perceived this development as unfair, because the 6 doctors available had to take on the work of 25 doctors.^29^

The consequences of unfair distribution of doctors included work overload, doctors feeling overused, complaints of ill health, tiredness and waning motivation. Some devised coping strategies including switching their phones off when off duty and refusing to visit some of the wards in the maternity department during ward rounds. Some placed quotas for the number of clients they would attend to in a day.^30^ The findings supports Manongi *et al.* (2006) and Mbindyo *et al.*’s (2009) studies, which suggest that health workers give quotas when they are overwhelmed with work. Others performed only emergency CS and skipped elective CS, while some left, giving the maternity a relatively high doctor turnover. An interview with Dr Job* who was described as a good doctor, but left Facility A depicts the process from feelings of injustice to demotivation to attrition.

“I got tired…It gets to a point you begin to feel that those managing the system don’t really care about those who are busily doing the work. So whether you go to work and there is no water or whether you go to work and the laundry is not functioning, whether you go to work and the unit that sterilizes the equipment is not functioning, whether you have enough medical officers or house officers to support you do the work or not, nobody seemed to be finding permanent solutions to these problems. So once in a while we run into different forms of crisis… and then you find out that you are getting more and more irritated with everybody who work with you. You snap at nurses, you snap at patients. You get up in the morning, particularly on the days that you are going on calls, you are not happy to be going to work.”^31^

A senior nurse manager explained that the limited number of doctors in the maternity department of Facility A was a national problem. She explained that there are quotas imposed on the number of workers that the GHS can employ at a given time. Second, the teaching hospitals, which are the training institutions that feed public hospitals with doctors, retain most of the doctors they train.^32^ The skewed distribution of doctors in low resource countries including Ghana has been noted elsewhere (Dovlo 1998; Agyepong *et al.* 2001; Chen *et al.* 2004; Snow *et al.* 2012; Faye *et al.* 2013; Mutale *et al.* 2013). Unfortunately, in many countries the establishment of posts, recruitment, terms and conditions of service are beyond the authority of public hospitals and regional managers. They are directly controlled by central government agencies (Larbi 1998, 2005; Appiah-Denkyira *et al.* 2011). Second per the GHS and Teaching Hospitals’ Act 525 (Government of Ghana 1996), the regional health directorate and the GHS have no authority over doctors in the teaching hospitals, which are a major source of recruitment of doctors and other frontline workers. These gaps are translated into skewed distribution of doctors in public health facilities as was the case in Facility A.

Frontline workers in the maternity department of both facilities perceived distributive injustice in national policy related to worker remuneration. They suggested that since they were attending to higher client numbers than their colleagues in other departments, they should be given incentives to make up for the low remuneration from government.^33^ A senior doctor in the maternity department of Facility A presented this view:

“Dr Kofi: The Ghana Health Service system is such that the physician specialist and the gynaecologist receive the same salary. But the physician specialist will come in the morning, do the prescriptions and by afternoon he is done… But our work is different; you can be called at any time .…sometimes they call me at 2:30 am.

Researcher: So do you think your midwives have a case when they complain that they are not being treated fairly?

Dr. Kofi*: Yes, their complaints are right. Because they work a lot, but are not given much…the problem is a national one. For instance workers of the same rank are given the same salary across the country. So a nurse of the same rank whether the fellow is in the labour ward, the out patients department or wherever receives the same salary.”^34^

A member of management in Facility A agreed that the quantum of work in the maternity department was comparatively higher than in the other departments, so the workers in the maternity department should be compensated for the extra work.^35^ However, a senior nurse manager in Facility A and two management workers in Facility B held the view that all departments are important, so they should be treated equally. The priority should be on using the IGFs to run the hospital and any surpluses could be used to provide incentives to motivate all workers.^36^

Another national policy issue cited by workers as unjust both from a distributive and procedural injustice perspective was the implementation of the fee free delivery policy, which involved universal exemptions from payment of user fees for delivery services (Ansong-Tornui *et al.* 2007). Frontline workers in Facility A suggested that the policy had led to an increased client load in the maternity department, without a corresponding increase in staff numbers, basic equipment, tools and supplies, worker remuneration and expansion of infrastructure. This was unfair. To use the words of one of the senior doctors:

“I am disgruntled and angry but we have to work. They refused to give us our conversion difference (salary adjustment).…Look at the clients; some are sitting on benches. Facility A, two thirds of the land has not been used, we have a large plot of land and what is being done with it! Look at the small thing they are putting up as the maternity block and look at how long it has taken.”^37^

Workers perceived that the national policy on the fee free delivery service had been implemented without taking into consideration the ability of facilities and workers to manage excess numbers or how to compensate workers for the extra work. The increase in numbers had put a strain on workers and facilities, which was demotivating. Similar finding have been reported (Ansong-Tornui *et al.* 2007). Other studies have documented frontline workers’ perceptions of unfair remuneration with agitations for better remuneration in Ghana (Agyepong *et al.* 2012). Songstad *et al.* (2011) also noted the influence of policy and political developments on worker remuneration and perceptions of injustice in Tanzania.

### Perceived interactional injustice related to hospital management

In Facility B, many frontline workers perceived interactional injustice from hospital management in meting out punishments and rewards and in communicating with workers. Frontline workers suggested that the head of the hospital did not commend them for good work done, but was quick to reproach (insult) workers who made mistakes. They found her approach to interacting with them unprofessional and demotivating.^38^ An interview with two management workers confirmed frontline workers’ perceptions about the head. The management workers added that if a worker made a mistake, the head of the facility insulted the worker and also insulted his or her entire family. Also if the worker in question ever made another mistake in future, the head always referred to her previous mistakes.^39^ A senior nurse said she had indicated in a staff survey questionnaire in 2013 that they were not commended for their good work, but were always reproached by the hospital management for shortcomings.^40^

The management workers who were interviewed as well as the frontline workers admitted that the head of the hospital had the right to discipline workers. However, they said they would have preferred an approach to discipline with the head appropriately investigating reported offences first, then dealing with the offences in a professional way, instead of making discipline seem like a personal attack on workers. They argued that dealing with offences in a professional manner could help bring long-term solutions and prevent recurrence of similar offences.^41^ In an interview with the researcher, the head of Facility B said that she follows the GHS code of ethics^42^ to discipline offending workers. This entails: she first gives a verbal warning to an offender, followed by a written warning and the third time she hands the offender over with the compiled evidence to the district health directorate or the regional health directorate for action. On the issue of workers complaining that she reproaches them for their offences, she explained that once a worker commits an offence, she reprimands the worker in her office in the presence of the worker’s department head who serves as a witness. However, if the fellow repeats a similar offence she refers the worker to the previous offence, because the worker would have probably promised to be of good behaviour, but might have forgotten and committed a similar offence.^43^

Some workers suggested that existing channels for communicating concerns to management were not helpful. A former management worker said they had durbars,^44^ which were not useful channels for communicating their concerns to management. He said frontline workers complained that in previous durbars when they raised their concerns, the head of the facility responded in an unfriendly manner. Consequently, very few workers attend durbars.^45^ The head of Facility B said in an interview that she did not see her responses at durbars as a confrontation; this was probably the perception of some workers. She stated that she and her core management team members make efforts to address workers’ concerns at durbars.^46^

Thus ironically workers felt that the professional work ethics that they were being espoused to hold for their clients, were not being reciprocated to them by the hospital management. Perceived interactional injustice contributed to feelings of bitterness, sorrow and anger, which affected some workers’ self confidence, interest and desire to perform their duties.^47^ Consequently, some workers did not take initiatives to facilitate health service provision to clients and sometimes counterproductive behaviours were observed. On one occasion women who had completed their antenatal visit could not leave the facility, because they had to take their drugs from the antenatal pharmacy. However there was no dispensary attendant, so the women sat waiting for another hour. The junior nurse who provided them with the antenatal service got worried and asked her superior if they could do anything about the women’s plight since there was no dispensary attendant at the dispensary to attend to them. The superior responded: ‘I don’t care what happens. If I talk then they will report me to doctor (head of Facility B). So I won’t bother myself.’^48^ Subsequently, the women who overheard her comment left the facility without waiting any longer to receive their routine antenatal drugs.

Workers’ perception of being treated with disrespect and in an insensitive manner contributed to poor organizational climate and lack of job satisfaction and the desire to leave the facility. Similar findings have been noted elsewhere (Laschinger 2003, 2004; Almost *et al.* 2010). Also, Mathauer and Imhoff (2006) found that appreciation of their work and recognition among others were important ingredients to worker motivation and a perceived sense of justice. Fonn and Xaba (2001) infer that when health system managers treat workers fairly respecting their rights, empowering them and creating a conducive work environment, workers become motivated and exhibit positive attitudes towards work.

#### Intrinsic motivation factors

Most frontline workers perceived injustice at hospital management and policy levels, which they suggested affected their motivation. However, interestingly some of these workers demonstrated a high sense of motivation and responded positively to clients’ needs in spite of this. In-depth interviews and conversations with some workers who were observed to exhibit a high sense of motivation suggested that the factors motivating them were intrinsic. Intrinsically motivating factors were similar in both facilities. Sources of workers’ intrinsic motivation included perceiving clients as human beings with rights and the desire to maintain standards and accountability to God for one’s actions. Others were a perception of their work as a contract that must be honoured, a strong sense of duty and the obligation to use their professional knowledge ethically. Some intrinsically motivated workers suggested that the greatest incentives to them included successful client recovery, which gave them an inner sense of satisfaction and others believed they received blessings from God for responding positively to clients’ needs. Below are illustrative excerpts from two workers. The first is a doctor in Facility A, whose motives were clients’ rights, a high sense of duty and a desire to maintain standards. The second is a nurse in Facility B whose motives included professional ethics and deriving inner satisfaction from successful outcomes.

“I don’t want to mismanage anyone. I don’t want to give half-half to anyone. I don’t want to see someone and it is like you are experimenting, no. If I see you, I want to give you the very best I can and standard treatment that you deserve. Not because you are in Ghana, so you don’t have this, no.… I don’t want to cut corners.”^49^

You see, I believe that when you are doing a job you have to do it well. When I came here (Facility B) the first time, I realized that there was no oxygen and I said I won’t work without oxygen. The then matron… had to get it before I became comfortable to work here. You know, when you are working, the inner satisfaction is very important. How can you deliver a mother and the baby needs resuscitation and you cannot do so and you watch the baby die.^50^

These two workers and several others like them who were intrinsically motivated exhibited positive attitudes including sacrificing to stay back to attend to clients past their scheduled times. In emergencies, some used their personal resources including going to other hospitals to beg for supplies for their hospital. Some improvized in the absence of critical supplies to save lives.^51^ The influence of intrinsic motivation on worker performance is consistent with Lin’s (2007) finding that workers’ attitudes and intentions to perform tasks are strongly associated with their intrinsic motivation. Studies in Benin suggests that vocation, professional conscience, job satisfaction and the desire to help clients are strong motivating factors for health workers (Mathauer and Imhoff 2006). Studies carried out in India found that intrinsic factors had a higher influence on doctors’ motivation in the provision of health care than extrinsic factors (Purohit and Bandyopadhyay 2014).

## Summary of findings

Our findings support studies that suggest that workers’ motivation is influenced by extrinsic and intrinsic factors. We found that perceptions of distributive, procedural and interactional injustice at organizational and policy levels had a strong influence on workers’ motivation and response to clients’ health care needs. Frontline workers had the feeling of being let down by the health system as they perceived that they did not receive ‘people-centred care’ from their employers, despite being asked to provide ‘people-centred care’ to the clients that come to their hospitals. They perceived that the values they are being asked to hold for their external customers are not being held for them by the health system within which they work. This considerably weakens the credibility of the message they are being given to treat their clients in a responsive manner. Furthermore, perceived injustice in policy and organizational processes made them distrust their leadership. Some became apathetic and less motivated to respond to external clients’ needs.

Despite perceived injustice in policy and organizational processes, some workers demonstrated a high sense of motivation and responded positively to clients’ health care needs. We found that intrinsic motivation factors including perceiving clients as human beings with rights, the desire to maintain standards and accountability to God for one’s actions among others, played a key role in workers who demonstrated a high sense of motivation. Intrinsically motivated workers suggested that they derived inner satisfaction from performing tasks and others believed that they received blessings from God for responding to clients’ needs. Nevertheless, even intrinsically motivated workers such as Dr Job* burned out with time. This shows that worker motivation is a dynamic process.

## Conclusion

Our methodology of a participatory approach through participant observation, conversations and in-depth interviews in studying frontline worker motivation in a biomedical environment provides insights on organizational justice in the hospital environment that could not have been otherwise obtained.

Using distributive, procedural and interactional justice dimensions of organizational justice theory, this study has demonstrated the multiple layers of injustice perceived by health workers in the hospital setting. It brings to light the influence of worker perception of injustice on worker motivation in the provision of health care. Where workers perceived injustice, workers were more likely to be demotivated and it affected their response to client health care needs. However, issues of injustice could not explain why some workers were motivated to respond to clients’ needs. Factors that were identified to motivate workers were intrinsic. Thus, this study contributes to knowledge on the complexity of factors that influence frontline worker motivation within the hospital setting.

To promote worker motivation a ‘people-centred care’ approach that considers frontline workers within health system as ‘people’ to whom the system should be responsive is essential. Health care should draw upon CQI philosophy and should be organized around health workers as internal customers and clients as external customers. Frontline workers’ interest should be factored into any intervention that aims at improving quality health care.

Within our study setting a ‘people-centred’ approach that includes frontline health workers in the concept should include the following:

At facility level, supportive leadership and supervision should be instituted to foster good working relationships between frontline workers and managers. There is a need to train managers in transparency, communication, respect in interaction and the need to see team work as a priority as proposed in the CQI philosophy.

At facility level a radical change in management culture is needed. Management should put in structures that will ensure effective communication, transparency and accountability. Also, managers and supervisors should learn to see workers as members of a team who should be treated with dignity and respect even in matters of discipline. Facilities should improve motivation through provision of basic incentives to frontline workers.

At national and regional levels efforts should be made to synchronize the needs of the various facilities to be able to distribute frontline workers based on need of facilities. Transparent processes for allocating workers that engage frontline workers and are seen as fair in the context of overall national resource constraints should be adopted.

We believe that without the creation of a conducive atmosphere where frontline workers will feel their concerns are that of their departmental organization managers, policy makers and other agents responsible for health care in a way that is fair, it will be difficult to have frontline workers motivated to see the health of their clients as their concern.

## Endnotes

^1^ Facility A: observation notes, May 24, 2012.

^2^ Facility A: conversation with a doctor, May 7, 2012; Conversation with a midwife, July 27, 2012; Interview with a nurse, September 20, 2012; Observation notes, May 24, 2012.

^3^ Facility A: conversation with an anaesthetist, July 5, 2012.

^4^ Interview with Dr *Bill, former worker of Facility A, September 9, 2013.

^5^ Facility A: interview with an accountant, August 17, 2012.

^6^ Facility A: conversation with a senior midwife, June 8, 12.

^7^ Facility A: interview with Hospital manager, December 4, 2013.

^8^ Facility B: Conversation with a nurse, August 22, 2012; Conversation with a nurse, July 29, 2013; Conversation with two night nurses, August 11, 2013.

^9^ An annual performance review of public hospitals in the Greater Accra Region. This was instituted by the regional health directorate to improve health care quality.

^10^ Facility B: interview with two management members, August 6, 2013; conversation with two nurses, August 11, 2013.

^11^ Facility B: conversation with two night nurses, August 11, 2013.

^12^ Facility A: observation notes, maternal audit meeting, March 16, 2013.

^13^ Facility A: conversation with an anaesthetist, July 5, 2012.

^14^ Facility A: conversation with an anaesthetist, October 3, 2013; Facility B: conversation with a nurse, September 26, 2012.

^15^ Facility A: conversations with two anaesthetists, October 3, 2013, September 26, 2013; Facility B: interview with senior nurse, July 30, 2013.

^16^ Facility A: interview with a management member, July 31, 2012.

^17^ Ocytocin is a drug commonly used in induction and argumentation of labouring clients (Freeman and Nageotte 2007).

^18^ Facility B: interview with two management members, August 6, 2013.

^19^ Facility A: conversation with an anaesthetist, July 5, 2012.

^20^ Locum is working in private facilities in addition to being permanent workers in public hospitals.

^21^ Facility B: conversation with a nurse, July 18, 2013; Interview with Dr Job, August 3, 2013.

^22^ Facility A: conversation with a junior doctor, July 23, 2012.

^23^ Facility A: observation notes, September 14, 2012.

^24^ Facility A: interviews with a management member, July 31, 2012; Facility B: interview with two management members, September 20, 2013.

^25^ Facility A: interview with a junior doctor, September 14, 2012.

^26^ Facility B: interview with senior nurse, July 30, 2013.

^27^ Interview with legal expert, September 8, 2013.

^28^ Facility A: observation notes, November 23, 2012.

^29^ Facility A: conversation with two gynaecologists, a junior doctor and a house officer, April 30, 2012; interview with Dr Job*, August 3, 2013.

^30^ Facility A: interview with a junior doctor, October 31, 2012.

^31^ Interview with Dr Job*, August 3,2013.

^32^ Facility A: conversation with senior nurse, November 26, 2013.

^33^ Facility A: conversation with two nurses, April 20, 2012.

^34^ Facility A: conversation with a senior doctor, July 5, 2012.

^35^ Facility A: interview with physician specialist, July 31, 2012.

^36^ Facility A: interview with a nurse, September 20, 2012; Facility B: interview with two management members, August 6, 2013.

^37^ Facility A: conversation with a senior doctor, October 3, 2013.

^38^ Facility B: interview with a doctor, July 29, 2013; interview with two nurses July 30, 2013; conversation with a nurse, July 29, 2013.

^39^ Facility B: interviews with two management members, August 6, 2013.

^40^ Facility B: interview with a nurse, July 30, 2013.

^41^ Facility B: interview with two management members, August 6, 2013; interview with a doctor, July 29, 2013; Conversation with a nurse, July 29, 2013.

^42^ The GHS code of conduct and disciplinary procedures stipulates how matters of workers discipline should be handled by health service managers. GHS 2003. Code of Conduct and disciplinary Procedures, Accra, Ghana.

^43^ Facility B: interview with hospital manager, March 5, 2014.

^44^ Open air meetings that brings together management and frontline workers to interact freely to discuss organizational issues.

^45^ Facility B: interview with former management member, August 7, 2012.

^46^ Facility B: interview with hospital manager, March 5, 2014.

^47^ Facility B: interview with a medical doctor, July 29, 2013; Interview with two nurses, July 30, 2013; conversation with a nurse, July 29, 2013.

^48^ Facility B: observation notes, July 18, 2013.

^49^ Facility A: interview with a junior doctor, September 14, 2012.

^50^ Facility B: conversation with a nurse, September 5, 2012.

^51^ Facility A: observation notes, July 3, 2012, September 17, 2012; Facility B: observation notes, November 1, 2012.
